# Physiological And Anaesthetic Considerations For The Preterm Neonate Undergoing Surgery

**Published:** 2012-01-01

**Authors:** Bharti Taneja, Vinish Srivastava, Kirti N Saxena

**Affiliations:** Department of Anaesthesiology and Intensive Care, Maulana Azad Medical College New Delhi 110002, India

With improvements in healthcare, the survival of preterm babies has increased significantly and these preterm and ex-preterm babies often present for various surgical interventions. Previously, it was believed that due to the immaturity of the nervous system, these preterm babies do not experience or feel pain and consequently inadequate or no anaesthesia was administered to them. Recent work has shown that as early as the 13th to 20th week of gestation, there is perception of pain. Evoked potentials and cerebral glucose metabolism offer evidence of functional maturity and responses such as changes in pulse and blood pressure, crying, and grimacing have been quantified for circumcisions done without anaesthesia [1]. Determination of gestational age is important to assess risks for morbidity and mortality in neonates.

In view of the immature physiology of the preterm and ex preterm neonate, these patients present a great challenge to the anaesthesiologist. The various factors that increase morbidity and mortality in these patients are usually consequent to the immaturity of the various body systems and the associated congenital defects.

**Central nervous system**

Peri/intra-ventricular (PVH-IVH) hemorrhage remains a significant cause of both morbidity and mortality in infants who are born prematurely. Sequelae of PVH-IVH include life-long neurological deficits, such as cerebral palsy, developmental delay, and seizures [2]. Because PVH-IVH can occur without clinical signs, screening and serial examinations are necessary for the diagnosis. The preterm neonate is at a greater risk for intracranial hemorrhage which may be due to changes in blood flow, blood pressure, and various other factors including serum osmolality [3].

**Respiratory system**

The respiratory bronchioles formation and production of surfactant begins by 24 weeks’ gestation, at which stage extra uterine survival is possible, though surfactant concentrations may remain inadequate until 36 weeks. The fetal lung structure and immature functional capacity are at greatest risk of respiratory distress, need for oxygen and positive-pressure ventilation, and admission for intensive care. These patients have a greater incidence of bronchopulmonary dysplasia (BPD) and chronic lung disease (CLD). Respiratory distress syndrome is caused by surfactant deficiency and is inversely related to gestational age affecting 90% of infants born at 26 weeks’ gestation. Anaesthesia may cause low lung volumes and ventilation/perfusion mismatch. The combination of immature structural development, disease and anaesthesia increases the chance of hypoxia and an anaesthetic technique based on mechanical ventilation with the use of positive end-expiratory pressure should avoid this complication. The risks of oxygen toxicity and baro­traumas should also be considered during anaesthesia and the goal should be to maintain adequate oxygenation and ventilation, with the minimum oxygen concentration and peak inspiratory pressure necessary [4-6].

**Apnoea of prematurity**

Apnoeic spell is defined as a pause in breathing of more than 20 seconds or one of less than 20 seconds associated with bradycardia and/or cyanosis [2]. The incidence of apnoea is related to the gestational as well as post conceptional age. An infant born at a lower post conceptional stage is more likely to have apnoea than one with same post conceptional age but born later. This risk is approximately 45% at 48 weeks post conceptional age up to 60 weeks. This risk reduces with increase in duration of post conceptual age upto 60 weeks post conceptional age [7]. Aponea in premature infants is exacerbated by hypoxia, sepsis, intracranial haemorrhage, metabolic abnormalities, hypo/hyperthermia, upper airway obstruction, heart failure, anaemia, vasovagal reflexes and drugs, including prostaglandins and anaesthetic agents [8]. Apnoeic spells are treated by stimulation, bag-mask ventilation, addressing underlying abnormalities, the use of respiratory stimulants such as caffeine or theophylline, neonatal CPAP or ventilation [9-11]. These infants therefore require careful post operative monitoring and HDU (High Dependency Unit) stay for at least 24hrs and should not be anaesthetized as outpatients even if regional anaesthesia has been administered. In fact some workers suggest that wherever possible, anaesthesia should be delayed until the ex premature infant is older than 52 weeks post conceptional age [12].

**Cardiovascular system**

The premature infant is at greater risk of cardiovascular compromise during anaesthesia and surgery than the term infant. They have poor diastolic function, the left ventricle is less compliant and cardiac output depends more on the heart rate than in the term infant. Premature babies have small absolute blood volumes, therefore relatively minor blood loss during surgery can rapidly lead to hypovolaemia, hypotension and shock. Autoregulation is not well developed in premature babies and the heart rate may not increase with hypovolaemia. Also, anaesthesia blunts the baroreflexes in premature infants, further limiting the ability to compensate for hypovolaemia [6].

There may be associated cardiac congenital defects. Patent ductus arteriosus (PDA) is almost a normal finding in the preterm ( >50% incidence) with significant left-to-right shunting and risk for the development of congestive heart failure, IVH, necrotizing enterocolitis (NEC), renal insufficiency, pulmonary edema, or hemorrhage due to PDA and retinopathy of prematurity (ROP). Various treatment strategies for closure of a PDA include careful fluid administration, diuretics and prostaglandin synthetase inhibitors (PSI) such as Indomethacin or Ibuprofen. Surgical PDA ligation is considered when medical treatment had either failed or is contraindicated [13-15].

**Temperature Regulation, Skin and Body Surface**

The epidermis is thinner in the preterm below 32 wks. As a result, they have increased susceptibility to damage from the most trivial trauma and tendency for greater fluid loss through the skin. Thermoregulation in the neonate is limited and easily overwhelmed by environmental conditions. There is a great potential for heat loss (high body surface area to body weight ratio, increased thermal conductance, increased evaporative heat loss through the skin) and limited heat production through brown fat metabolism. The preterm baby is particularly vulnerable as the immature skin is thin and allows major heat (and evaporative fluid) losses. The principle of anaesthesia in these infants is for minimal handling in a warm environment [2].

**Haematologic system**

Newborns born at 26 to 28 weeks have lower hemoglobin levels (13-15gm/dl) than term newborns, 70-80% of which maybe fetal haemoglobin. The blood volume is 100 to 110ml per kg. Preterm newborns are deficient in both vitamin K and vitamin K–dependent coagulation factors and may also have a relatively low platelet count [16-18].

**Renal function**

Functional capacity is related to gestational age. The limited capacity of renal tubules to reabsorb bicarbonate accounts for the ‘normal’ acidosis seen in newborns. The ability to retain sodium effectively does not develop until 32 weeks. The distal tubular response to aldosterone is low until 34 weeks and antidiuretic hormone levels in the premature neonate are high. These factors predispose to hyponatraemia. The total body water in the preterm infant is higher than in a term neonate (75–85% of body weight) and is inversely related to the gestational age. The marked transepidermal permeability due to skin immaturity and a relatively large body surface area leads to 15-fold increase in the evaporative water loss during the first few days of life as compared with term babies [19].

**Gastrointestinal function**

Necrotizing enterocolitis (NEC) with bowel wall necrosis and possible perforation can occur in these babies, which can further lead to systemic sepsis. Bowel perforation may be the result of administration of indomethacin and corticosteroids. Gastro-oesophageal reflux is common, not only because of neurological immaturity but also results from an underdeveloped and incompetent lower oesophageal sphincter leading to laryngospasm, laryngitis, tracheitis, apnoea, chronic cough, otitis media, and asthma. It should be treated aggressively to minimize upper and lower respiratory complications in these populations who are already predisposed to pulmonary dysfunction [19,20].

**Glucose homeostasis**

Liver stores of glycogen (and iron) are made up primarily in the last trimester of fetal life and consequently preterm neonates have very limited glycogen stores. Also, ketogenesis and lipolysis capability are limited. It is therefore critical to carefully monitor serum glucose during anesthesia more so because all the clinical signs of hypoglycemia may not be evidenced during general anaesthesia. Currently we know that preterm newborns require the same glucose levels as term infants. Hyperglycaemia should be avoided as a hyperosmolar state can lead to intraventricular haemorrhage (IVH), osmotic diuresis, and dehydration [19,20].

The other factors that increase morbidity and mortality in the preterm are the associated congenital anomalies and considerations regarding use of high concentrations of oxygen.

**Anaesthetic management of the preterm neonate**

The anaesthesiologist is usually called to administer anaesthesia in the preterm for the following situations:

- PDA ligation (20–30% incidence in moderately premature neonates)
- Laparotomy for NEC or spontaneous bowel perforation
- Laparotomy for reanastomosis of bowel or relief of adhesions
- Inguinal hernia repair (2% incidence in females and 7–30% incidence in males with 60% risk of incarceration)
- Fundoplication for unresolving and symptomatic oesophageal reflux
- Vascular access under X-ray control
- Viterectomy or laser surgery for retinopathy of prematurity
- CSF drainage or ventriculoperitoneal shunt insertion for obstructive hydrocephalus after intraventricular haemorrhage
- CT and MRI scanning to evaluate cerebral damage

The most commonly performed surgery is the repair of inguinal hernia, appearing in 38% of infants whose birth weight is between 751g and 1000g and in 16% of those whose birth weight is between 1001g and 1250g. Inguinal hernia require early surgical repair because of the risk of bowel incarceration and vascular compromise in bowel and gonadal tissue [21,22]. In addition, preterm newborns may be born with the other coexisting congenital anomalies as term infants that require urgent or emergent surgery such as intestinal atresia, gastroschisis, omphalocele, and esophageal atresia with or without tracheoesophageal fistula or congenital diaphragmatic hernia.

**Preanaesthetic Evaluation**

Preoperative assessment should include parental consultation with the description of a likely deterioration in pulmonary function necessitating postoperative ventilatory support. The infant's antenatal history, gestational age, and weight are prerequisite information for surgical procedures [23]. A detailed complete system examination should be carried out with special reference to upper airway appearance and possibility of difficult intubation.

The routine investigations - Haemoglobin, haematocrit, platelets, and the coagulation profile should be within acceptable limits. Preoperative serum electrolyte and glucose levels will help guide requirements in theatre. An ABG, X-ray chest and an echocardiogram are desirable prior to surgery. Cross-matched blood must be available so that transfusion can be given whenever the blood loss is more than 10% of blood volume during the surgery [20].

Periods of preoperative fasting must be taken into account to calculate fluid deficits that need replacement intraoperatively. Neonates undergoing elective surgery can be fed until 4 hours before anaesthesia and then given clear fluids until 2 hours before surgery. Breast milk and Formula feeds are considered in the same category as solids [19]. If surgery is delayed for extended periods, IV fluids must be given, or if time permits, additional clear oral fluids are given. Some babies may be receiving total parenteral nutrition to provide their maintenance fluid, glucose, and electrolyte requirements. A detailed preanaesthetic check should be performed with special reference to detection and correction of fluid and electrolyte deficits.

Premedication is usually avoided as there may be a dramatic respiratory depressant response to the drugs. However, atropine (10–20 mg kg) can be considered to preempt transient bradycardia, although these are less frequent during anaesthesia with newer inhalation agents. All babies should receive vitamin K before operation.

**Practical Points for the conduct of anaesthesia in a preterm neonate**

Some modifications for the conduct of anaesthesia may be required for these very tiny patients.

Monitoring

SpO2: Two pulse oximeters should be used; the first saturation probe is placed on the right hand (pre-ductal) and the other on a lower limb (post-ductal). These oximeters should be of the “neonatal” variety, with less adhesive to minimize skin damage.

Blood pressure: Proper size and application is important since repeated noninvasive blood pressure measurement can fracture very poorly ossified, calcium-deficient bones. The definition of a normal systemic blood pressure is problematic in the extremely low gestational age newborn. A safe “rule of thumb” is to treat the mean arterial blood pressure if it is below the gestational age + 5 mm Hg. Treatment options are variable and include IV fluid, or vasopressors [24,25].

ECG: Adhesive on electrocardiogram (ECG) stickers can damage the skin. Removal of adhesive tape or monitors can damage the skin akin to a partial thickness burn. After delivery, the skin of preterm newborns does mature rapidly and achieves greater thickness within a few weeks

EtCO2: Because of very small tidal volumes and low maximum expiratory flow rate, end-tidal CO2 measurement will be rendered less accurate but is important nonetheless [24].

IBP: Invasive blood pressure or central venous pressure measurement, although desirable, is so technically challenging and potentially dangerous that often these are not employed. If an umbilical artery line is in place, rapid sampling and flushing can lead to disastrous consequences [24].

Temperature monitoring: It is important and intraoesophageal temperature can be monitored using a combined stethoscope and temperature probe. But this may itself pose a risk as sometimes perforation is possible despite meticulous and careful placement of rectal or esophageal probes [24].

OT preparation

Staffing: Extra personnel and help may be required. An experienced operating department practitioner is essential.

Equipment: All equipment including a Mapleson F circuit for hand ventilation, anaesthetic machine, ventilator, breathing circuits, infusion pumps, and laryngoscopes should be prechecked. Electrical supply for infusion pumps must be assured, especially if inotropic support is to be continued. A pressure controlled ventilator capable of delivering small tidal volumes with PEEP is essential.

Airway: Securing the airway is difficult in these very tiny babies and proper-sized equipment and endotracheal tubes and masks should be available and ready. These patients generally arrive to the OT already intubated, thus the anaesthetic plan should include post­operative mechanical ventilation. A “000” face mask, “0” Miller blades and 0-sized laryngeal mask airways (LMAs) should be available, as well as endotracheal tubes of 2.5, 3.0 and 3.5 mm outer diameter (OD) with appropriately sized stylets.

OT temperature: Premature infants have a greater tendency for heat loss. The ambient OT temperature must be kept around 27 C. Patient should be kept warm by using a warming mattress and a warm air blanket. In­spired gases should be heated and humidified with a paediatric heat-moisture exchanger. I.V. fluids, blood, and blood products should be warmed and used. Irrigation fluid must be warmed. Other ways to keep the patient warm include application of the waterproof surgical drape to the non-involved areas.

Positioning: The majority of preterm neonates requiring emergency surgery are likely to be intubated and ventilated and transported in a dedicated transfer incubator. The baby must be carefully but expeditiously transferred to the operating table taking care to avoid accidental disconnections and decannulations. In the smallest of neonates and especially when 360 access to the patient is required by the surgeon (e.g. laser surgery for retinopathy), access can be improved by removing the head and foot ends of the operating table.

**General anaesthesia**

Traditionally, general anaesthesia (GA) has been administered in the preterm and ex preterm neonates. The advantages of GA are better control over the respiratory and cardiovascular parameters.

There is considerable controversy currently over the long-term CNS effects of many anaesthetic medications such as midazolam, nitrous oxide, isoflurane and ketamine. There have been reports of choreaform movements with long-term administration of midazolam in newborns, and for this reason many NICUs no longer use this medication to sedate preterm newborns receiving mechanical ventilation [2,24].

Moderate concentrations of volatile agents can be used to minimize increase in PVR and to avoid decrease in systemic arterial pressure. Also inhaled nitric oxide can be used for the same. Although halothane has shown to cause postoperative complications, the newer shorter acting agents, desflurane may be particularly useful for recovery in a preterm infant prone for respiratory depression and apnoea [20,26,27].

Rapid sequence inductions can be challenging as even a short period of apnoea can cause detrimental desaturations, and mechanical pressure over the cricoid area can render the anatomy unrecognizable. Its use in children is questioned in contemporary practice. The tracheal length is 4 cm in a preterm neonate; therefore, the tube length should be carefully adjusted and secured. As a guide 1, 2, 3, and 4 kg babies should have the TT positioned at the gum margin at the 7, 8, 9, and 10 cm marks. The TT should allow a positive pressure breath with a small leak at 20–25 cm H2O pressure. Opioids have a long record of safety in preterm and fentanyl is advised as part of balanced anaesthesia in those with potential or overt pulmonary hypertension on ventilator therapy [20,28,29].

There is concern that even brief exposure to high oxygen levels is associated with increased morbidity and mortality in premature infants; fluctuations in oxygen levels should be avoided and oxygen saturation maintained between 88-95%, not exceeding 95%. Newborn resuscitation should be carried out with room air rather than 100% oxygen [30].

Tachycardia and hypertension can be detrimental in the presence of underdeveloped cerebral auto regulation. And hence careful titration of anaaesthetic and narcotic analgesic agents is necessary. For those who will be extubated after operation, minimal use of opioids combined with local anesthetic techniques and acetaminophen is beneficial, while planned postoperative ventilation indicates an opioid-based technique.

Sale et al, in their study on preterm infants under 37 weeks gestation and under 47weeks post conceptional age undergoing inguinal herniotomy suggested that light GA with sevoflurane or desflurane , controlled ventilation and caudal block is the current best technique for high risk preterm neonates [31].

In these patients, hypoglycemia should be anticipated and managed accordingly at a rate of 8 to 10 mg/kg/minute. This can be achieved by administering 10% dextrose at a rate of 110 to 120 ml/kg/day [32]. Glucose levels should be checked frequently, either by finger-stick using a glucometer or as part of an arterial blood gas measurement.

The blood volume of an 800-g newborn is 95 to 100 ml. Even what appears to be a small amount of bleeding is significant to such a small patient. Replacement of blood products must be done promptly but also slowly [33,34]. A 10-mL syringe of packed cells (PBRC) rapidly delivered into the vascular system over less than 1 minute will increase the blood volume by 10%. This is analogous to administering 2 units of PRBC to an adult over 1 minute. It is also very important that the blood products be warmed to minimize hypothermia in these patients.

**Regional anaesthesia**

In view of the high incidence of postoperative apnoea in preterm infants undergoing anaesthesia, a number of investigators have postulated a safe operating period that does not commence until a postmenstrual age of greater than 44 weeks, or even greater than 60 weeks. In addition to a suggested safe period of operation, awake regional anaesthesia has been suggested to reduce the incidence of post-operative apnoea, with an even lower incidence observed when no sedation is used. Prophylactic caffeine has also been used to prevent postoperative apnoea following general anaesthesia [35,36].

Studies have indicated that regional anaesthesia is not to be considered as a sole anaesthesia method for premature infants, but is beneficial as a supportive element when combined with GA. Smaller fractions of GA are required when regional anaesthesia is used along with it. Titration is recommended to ensure maximum effectiveness using minimal amounts of anaesthesia. In this category, as for GA, a cardiovascular complication is common, regardless of the combination used [37,39].

Regional anaesthetic techniques are popular due to the lack of exposure to volatile agents, omission of use of opiates in the postoperative period, uncomplicated execution and guarantee of excellent surgical conditions especially for inguinal hernia repair [6,40].

Preterm neonates can be managed successfully with spinal, caudal or combined spinal/caudal block for surgery. Regional techniques without sedation have been reported to have a reduced incidence of postoperative respiratory complications and may be more suitable than GA in the very young. However spinal anaesthesia in a preterm may be technically more difficult. In the study by Cavern et al, there were more technical failures in the spinal group. For every seven infants having a spinal anaesthetic, one failed to achieve accurate placement of the spinal needle. For every three patients having a spinal anaesthetic, one experienced an anaesthetic agent failure, requiring additional anaesthetic agent [6,39].

Spinal anaesthesia has been used successfully for inguinal hernia surgery in the preterm and ex pretem neonate and has shown to reduce the incidence of postoperative adverse events. Due to a larger volume of CSF and the increased surface area of nerve roots, a higher dose of local anaesthetics are required and the duration of action is also lesser as compared to older children and adults [35,41,42]. However haemodynamics are maintained by the low vasomotor tone and decreased vagal activity.

Caudal epidural anaesthesia can be employed safely and effectively either as single bolus dose or in the continuous form, via catheter. The advantage of using epidural catheter is that the level and duration of anaesthesia can be extended. Also lower volume of drugs can be used [42-44].

Gunter et al, evaluated the efficiency of several concentrations of bupivacaine (0.125 to 0.25%) for caudal anaesthesia in patients between 1 and 8 years of age, submitted to inguinal herniotomy. In this study, all the concentrations proved to be effective when associated with general inhalation anaesthesia with halo­thane and nitrous oxide. Caudal anaesthesia has been used successfully with light GA with newer inhalation agents [31,45].Various other blocks like ilio-inguinal nerve blocks have been used successfully. Topical anaesthetics have been used to reduce pain in premature infants during eye examination for retinopathy of prematurity [46].

**Post operative analgesia**

The aims of postoperative analgesia are to recognize pain, to minimize moderate and severe pain safely, to prevent pain where it is predictable, to bring pain rapidly under control and to continue pain control after discharge from hospital [47]. Pain is prevented using multi-modal analgesia, based on four classes of analgesics, namely local anaesthetics, opioids, non-steroidal antiinflammatory drugs (NSAIDs), and acetaminophen (paracetamol).

Opioids, because of their dramatic response on the respiratory system and tendency to cause postoperative apnoea, are not recommended for post operative analgesia in preterm and ex-preterm neonates. Local anaesthetics, NSAIDs, and acetaminophen (paracetamol) are generally preferred. However, most NSAIDS are not suitable for use in infants less than 6 months of age. Paracetamol is the only analgesic that can be safely used for postoperative analgesia in preterm in­fants [47-48].

In particular, a local/regional analgesic technique may be employed for all surgical cases unless there is a specific reason or an indication not to do so. Various field blocks like Ilioinguinal block for hernia surgery and Intercostal nerve blocks for PDA ligation can be used to supplement analgesia and manage postoperative pain in the preterm neonate [2].

**Conclusion**

Anaesthetizing a preterm neonate requires constant vigilance, rapid recognition of events and trends, and swift intervention.

The anaesthetic considerations in the preterm neonate are based on the physiological immaturity of the various body systems, associated congenital disorders, poor tolerance of anesthetic drugs and considerations regarding use of high concentrations of oxygen.

Clearly, the benefit of providing adequate anaesthesia and analgesia must be carefully balanced against the significant risk of cardio respiratory depression in this fragile population.

Titration of anaesthetics to a desired effect, while carefully monitoring the cardio respiratory status, is the goal. Premature and ex-premature infants may have dramatic responses to narcotics and potent inhaled anaesthetics. They have wide variability in their responses to anaesthetics and sedatives, and frequently experience exaggerated and unpredictable effects not observed in older infants and children.

Recently, the use of regional anaesthesia has shown to be safe and effective. It can reduce the complications and various adverse events generally associated with GA.

## Footnotes

**Source of Support:** Nil

**Conflict of Interest:** None declared

